# ER stress selects for *Candida auris* survivors with acquired fluconazole resistance and caspofungin tolerance

**DOI:** 10.3389/fmicb.2026.1845986

**Published:** 2026-09-25

**Authors:** Feng Yang, Liuliu Sun, Hao Li, Ting Fang, Qinli Guo, Nan Chen, Yuanying Jiang

**Affiliations:** Key Laboratory of Pathogen-Host Interaction (MOE), Department of Pharmacy, Shanghai Tenth People's Hospital, School of Medicine, Tongji University, Shanghai, China

**Keywords:** antifungal resistance, antifungal tolerance, *Candida auris*, ER stress, Hsp90-calcineurin axis

## Abstract

Exposure to endoplasmic reticulum (ER) stress can drive the emergence of antifungal resistance in pathogenic fungi, but the underlying mechanisms remain poorly understood. Here, we show that treatment of *Candida auris* B12037 (clade 3) with the ER stressor tunicamycin selects for survivors with acquired resistance to fluconazole and, in a subset, tolerance to caspofungin. Pharmacological inhibition revealed that caspofungin tolerance, but not fluconazole resistance, requires the Hsp90-calcineurin axis. RNA-seq analysis of the parental strain exposed to tunicamycin uncovered a coordinated stress response characterized by up-regulation of cell wall biosynthesis, transmembrane transport, and autophagy, alongside global repression of translation and energy metabolism. In the resistant survivors, fluconazole resistance correlated with up-regulation of the efflux pump *MDR1*, with isolate #10 additionally exhibiting *ERG11* overexpression. Caspofungin tolerance in isolate #10 was associated with up-regulation of multiple chitin synthase genes (*CHS1*, *CHS3*, *CHS5*), suggesting chitin-mediated cell wall remodeling. Isolate #19 displayed a distinct pattern, indicating alternative mechanisms of caspofungin tolerance. These findings reveal that ER stress selects for *C. auris* survivors with divergent antifungal resistance profiles through distinct molecular pathways, highlighting the potential for stress-induced evolution of multidrug resistance in this critical pathogen.

## Introduction

Since its first description in 2009, *Candida auris* has rapidly emerged as a formidable global health threat (Satoh et al., 2009). Unlike other *Candida* species, *C. auris* exhibits a unique combination of traits that complicate infection control and treatment. It colonizes human skin efficiently, persists on hospital surfaces for extended periods, and spreads readily between patients, leading to numerous healthcare-associated outbreaks across six continents (Eix and Nett, 2025). Importantly, bloodstream infections caused by *C. auris* are associated with mortality rates exceeding 40%, often compounded by delayed identification and limited therapeutic options (Athavale-Wad et al., 2025). The U.S. Centers for Disease Control and Prevention (CDC) has classified *C. auris* as an “urgent antimicrobial resistance threat” (Centers for Disease Control and Prevention, 2019), and the World Health Organization (WHO) has designated it as a “critical-priority” human fungal pathogen (WHO, 2022), underscoring the critical need to understand the mechanisms driving its resistance evolution.

Antifungal resistance in *C. auris* is multifaceted and often involves co-resistance to multiple drug classes. Resistance to azoles, the most frequently used antifungals, commonly arises from overexpression of efflux pumps, particularly *MDR1* (encoding a major facilitator superfamily transporter) and *CDR1* (encoding an ATP-binding cassette transporter), or from point mutations in the target gene *ERG11*, which encodes lanosterol 14α-demethylase (Marichal et al., 1999; Coste et al., 2004; Barker et al., 2025). For echinocandins, including caspofungin, resistance typically involves mutations in the *FKS1* gene, which encodes the catalytic subunit of β-1,3-glucan synthase (Doorley et al., 2025; Misas et al., 2025). However, clinical isolates lacking *FKS* mutations can also exhibit reduced echinocandin susceptibility through compensatory cell wall remodeling, such as increased chitin synthesis (Sharma et al., 2022). The coexistence of these mechanisms within single isolates can lead to pan-resistant phenotypes, leaving few or no effective treatment options.

Amphotericin B, a polyene antifungal that targets ergosterol in the fungal cell membrane, remains an important alternative for *C. auris* infections—particularly for neonatal cases, echinocandin-resistant isolates, and patients failing echinocandin therapy (Centers for Disease Control and Prevention, 2025). However, amphotericin B resistance has been documented in *C. auris*, with prevalence varying by clade and geographic region. Mutations in sterol biosynthesis genes, including *ERG2*, *ERG3*, *ERG6*, and *ERG11*, have been implicated in acquired amphotericin B resistance (da Silva et al., 2025; Massic et al., 2025).

Globally, six distinct clades of *C. auris* have been identified with varying antifungal susceptibility patterns and geographical distributions (Eix and Nett, 2025). Clades I (South Asia), III (Africa), and IV (South America) stand out due to their multidrug resistance, particularly to fluconazole and amphotericin B (Vaccaro et al., 2026). A comprehensive analysis of 1,031\u00B0*C. auris* strains revealed that clades I and III display the highest fluconazole resistance rates, with 94 and 96% of isolates resistant, respectively. In contrast, clades II, V, and VI have lower resistance rates, with clade VI being entirely susceptible to fluconazole (da Silva et al., 2025). Clade-associated point mutations in ERG11—including Y132F and K143R in clade I, and F126L in clade III—have been linked to azole resistance (Kappel et al., 2024). These clade-specific differences highlight the genetic and phenotypic heterogeneity of this pathogen and underscore the need for clade-informed interpretation of susceptibility data.

Antifungal resistance and tolerance represent two distinct but clinically relevant phenotypes that can compromise the efficacy of antifungal therapy. Resistance, quantified by the minimum inhibitory concentration (MIC), manifests as the capacity for growth at drug levels that suppress susceptible isolates. In contrast, antifungal tolerance is defined as the ability of drug-susceptible strains—frequently a subpopulation of cells—to exhibit slow growth under inhibitory concentrations. Importantly, tolerance is distinct from resistance and has been associated with persistent candidemia, suggesting that it may help predict patient responses to antifungal therapy [reviewed in Berman and Krysan (2020)]. In disk diffusion assays (DDAs), these phenotypes can be quantitatively distinguished using computational pipelines such as *diskImageR*, where the extent of resistance is indicated by the radius of inhibition (RAD), and tolerance by the fraction of growth (FoG) within the inhibition zone. Typically, a 20% drug inhibition threshold, corresponding to RAD₂₀ and FoG₂₀, is employed to evaluate resistance and tolerance, respectively (Gerstein et al., 2016; Rosenberg et al., 2018).

The evolution of azole tolerance has been extensively characterized in *C. albicans*. Exposure to high concentrations of fluconazole and posaconazole primarily drives the development of tolerance, while long-term exposure to subinhibitory fluconazole concentrations predominantly selects for resistance and, to a lesser extent, tolerance. In addition, tolerance to azoles can evolve independently of resistance and is frequently associated with aneuploidy (Kukurudz et al., 2022; Sun et al., 2023b; Todd et al., 2023; Yang et al., 2023). Although azole tolerance has also been documented in wild-type *C. auris* isolates, the selective pressures and mechanisms underlying this phenotype remain poorly understood (Rasouli Koohi et al., 2023).

The endoplasmic reticulum (ER) stress response, mediated by the unfolded protein response (UPR), has emerged as an important modulator of antifungal susceptibility in several pathogenic fungi. The link between ER stress and antifungal resistance has been documented across multiple fungal species. In *C. albicans*, exposure to TUN induces chromosome 2 trisomy, which not only confers tolerance to TUN itself but also cross-tolerance to caspofungin, a first-line echinocandin antifungal. This aneuploidy-driven tolerance involves overlapping stress response pathways, including protein kinase C (PKC) and calcineurin signaling (Yang et al., 2021a). In *Cryptococcus neoformans*, tunicamycin-induced ER stress—whether mild or severe—leads to the formation of aneuploid strains with diverse karyotypes, some of which confer resistance or cross-resistance to fluconazole and 5-flucytosine. Similarly, brefeldin A (BFA), another ER stress inducer, promotes aneuploidy formation in *C. neoformans*, primarily disomy of chromosomes 1, 3, and 7, with chromosome 1 disomy conferring cross-resistance to fluconazole and 5-flucytosine (Zhang et al., 2024; Zheng et al., 2024a). In *C. glabrata*, tunicamycin stress drives two distinct adaptive strategies: mitochondrial dysfunction (petite formation) and chromosome C aneuploidy, both of which confer cross-resistance to fluconazole (Dong et al., 2025). Beyond the *Candida* and *Cryptococcus* genera, the UPR has also been shown to play a critical role in virulence and antifungal susceptibility in the filamentous fungal pathogen *Aspergillus fumigatus*, where disruption of the UPR regulator hacA increases susceptibility to membrane- and cell wall-targeting antifungals (Richie et al., 2009). Collectively, these studies—spanning *C. albicans*, *C. glabrata*, *C. neoformans*, and *A. fumigatus*—suggest that ER stress adaptation can predispose diverse fungal pathogens to acquire resistance or tolerance to clinically important antifungals, often through aneuploidy-driven genomic plasticity or UPR-mediated stress adaptation.

Despite the well-documented roles of ER stress in modulating antifungal susceptibility in other fungal species (Richie et al., 2009; Yang et al., 2021a; Zhang et al., 2024; Zheng et al., 2024a; Dong et al., 2025), its impact on the emergence of resistance in *C. auris* has not been investigated. Given the exceptional capacity of *C. auris* to develop multidrug resistance and the central role of the UPR in integrating stress signals with cell wall integrity and efflux pump regulation, we hypothesized that exposure to ER stress could select for survivors with altered susceptibility to fluconazole and caspofungin. In this study, we addressed this hypothesis by subjecting *C. auris* B12037 (clade 3) to TUN-induced ER stress and characterizing the resulting survivors. Using a combination of phenotypic susceptibility testing, pharmacological inhibition of stress signaling pathways, and comparative transcriptomic analysis, we identified survivors with divergent resistance profiles. Our results reveal that ER stress selects for *C. auris* survivors with acquired resistance to fluconazole and tolerance to caspofungin through distinct molecular pathways, highlighting a previously unrecognized mechanism by which this critical pathogen may adapt to antifungal pressure. These findings have important implications for understanding the evolution of multidrug resistance in *C. auris* and may inform strategies to prevent its emergence.

## Materials and methods

### Strains and grow conditions

The *C. auris* clade III isolate B12037 was used as the wild-type strain. Stock cultures were preserved in 25% glycerol and stored at −80 °C. For routine growth, cells were cultured in yeast extract–peptone–dextrose (YPD) medium (1% [w/v] yeast extract, 2% [w/v] peptone, 2% [w/v] D-glucose) at 30 °C with shaking at 150–200 rpm. Solid media were prepared by adding 2% (w/v) agar to YPD.

### Spot assay

Cells were suspended in distilled water and adjusted to 1 × 10^7^ cells/mL; 3 μL of 10-fold serial dilutions were spotted on YPD with or without TUN (control). Plates were incubated at 30 °C and photographed after 2 days.

### Obtaining tunicamycin survivors

Cells were suspended in distilled water and adjusted to 1×10^7^ cells/mL.100 μL of the cell suspensions were spread on YPD plates supplemented with TUN (0, 2, 4, 8 and 16 μg/mL). The plates were incubated at 30 °C for 5 days. Adaptors were randomly chosen, streaked to YPD plates and incubated at 30 °C for 36 h. For each adaptor, several colonies with similar sizes were selected and frozen at −80 °C.

### Disk diffusion assay

The CLSI M44-A2 guidelines for antifungal disk diffusion susceptibility testing (Clinical and Laboratory Standards Institute, 2009) were followed with slight modifications. Briefly, strains were streaked from glycerol stocks onto YPD-agar and incubated for 48 h at 30 °C. Colonies were suspended in distilled water and adjusted to 1×10^6^ cells/ml. 100 μL of cell suspension were spread onto 15 mL SD-agar plates. An empty paper disk (6 mm diameter, and 0.7 mm thickness) supplemented with drug was placed in the center of each plate. Plates were then incubated at 30 °C and photographed at 48 h.

### RNA-seq

Test strains were cultured in YPD broth until reaching the logarithmic phase (OD_600_ = 1.0). To assess the effect of TUN on gene expression, the logarithmic phase cultures were split into two groups. One group received 4 μg/mL TUN supplementation, while the other was supplemented with an equivalent amount of DMSO (vehicle). After a 3-h incubation period, the cells were harvested by centrifugation. To compare gene expression between parent and survivors, log phase cultures were harvested by centrifugation.

Total RNA extraction and purification, library construction, and sequencing were performed as described previously (Sun et al., 2023a). Briefly, total RNA was extracted using the YeaStar™ RNA Kit (Zymo Research). RNA concentration, integrity, and purity were assessed using a Qubit 2.0 Fluorometer (Life Technologies), a Bioanalyzer 2,100 system (Agilent Technologies), and a NanoPhotometer (IMPLEN), respectively. Libraries were prepared with the VAHTS mRNA-seq v2 Library Prep Kit (Vazyme) and sequenced on an Illumina platform by BGI Genomics (Beijing, China). Raw reads underwent quality control with FastQC and were mapped to the *C. auris* clade III reference genome^1^ with the corresponding annotation file.

Three biological replicates were obtained for each strain. Differential gene expression profiling was carried out using DESeq2 (Love et al., 2014) with standard parameters. Genes with FDR (False Discovery Rate)-adjusted *p* value <0.05 and expression fold changes of more than 2 or less than 0.5 were considered differentially expressed.

### Rhodamine 6G efflux

Strains were revived from −80 °C glycerol stocks on YPD agar plates. Single colonies were inoculated into YPD broth and adjusted to an OD_600_ of 0.1, followed by incubation until the culture reached an OD_600_ of 1.0. Cells were harvested, washed, and resuspended in phosphate-buffered saline (PBS) to a final density of 1.0 × 10^8^ cells/mL. To deplete endogenous glucose, the cell suspensions were incubated at 30 °C for 1 h with shaking. Rhodamine 6G (Sigma-Aldrich, St. Louis, MO, United States) was then added to a final concentration of 10 μM, and the cultures were incubated at 30 °C for 30 min with shaking. After three washes with ice-cold sterile PBS, cells were resuspended in PBS containing 20 μM glucose to initiate efflux. Aliquots were collected at 0, 20, 40, 60, and 80 min post-glucose addition, and the fluorescence of the supernatant was measured using a Thermo Scientific Varioskan Flash microplate reader with SkanIt software (excitation at 527 nm, emission at 555 nm). All experiments were performed in three independent biological replicates.

### Nile red efflux assay

Nile red efflux was assessed following the protocol described by Toepfer et al. (2023). Briefly, cells were cultured overnight in 5 mL of YPD broth at 30 °C with agitation at 200 rpm. An inoculum from this culture was then diluted into 15 mL of fresh YPD broth to a starting OD_600nm_ of 0.5 and grown for until the OD_600nm_ reached about 1.0. Harvested cells were subjected to three washing steps, each using 10 mL of ice-cold PBS, with centrifugation at 3900 g for 5 min, after which they were kept on ice overnight. Prior to the assay, cells were allowed to equilibrate to room temperature over 30 min and then resuspended in 10 mL of 50 mM HEPES buffer adjusted to pH 7.0 using 0.1 M NaOH. To deplete cellular energy stores, cells were treated with 5 mM 2-deoxy-D-glucose (2-DOG) for 30 min at 30 °C under gentle shaking (50 rpm). Subsequently, Nile red was added to a final concentration of 7.5 μM, and the cells were incubated for another 30 min at 30 °C. Following two washes with ice-cold HEPES buffer, the samples resuspended in 15 mL HEPES buffer, and were maintained on ice for 2 h. For the efflux assay, flat-bottom 96-well plates were first filled with 50 μL of HEPES buffer per well, to which 100 μL of the Nile-red-loaded cell suspension was added. To trigger efflux, 50 μL of 80 mM glucose was added, while control wells received an equal volume of HEPES buffer instead. Fluorescence readings were taken on a plate reader with excitation at 485 nm and emission at 528 nm, recorded every 1 min over a 10-min period. Final Nile red fluorescence values were normalized and expressed as a percentage of the initial reading at time zero (T0).

### Measurement of cell wall chitin content

Chitin content was measured by quantifying acid-hydrolyzed glucosamine from purified cell walls following a previously described protocol (Yang et al., 2013). Briefly, strains were grown in YPD broth from OD_600_ 0.1 to 1.0, harvested, washed three times with sterile distilled water, and disrupted with 0.5-mm glass beads in a Mini-Beadbeater (BioSpec Products). Cell debris was washed five times with 1 M NaCl, and wall material was extracted by boiling in SDS-MeOH buffer (50 mM Tris, 2% SDS, 0.3 M β-mercaptoethanol, 1 mM EDTA, pH 8.0) at 100 °C for 10 min. Extracts were washed three times with sterile water and dried in a SpeedVac.

Approximately 4 mg of dried cell wall material was hydrolyzed in 1 mL of 6 M HCl at 100 °C for 17 h. After acid removal at 65 °C, the hydrolysate was reconstituted in 1 mL of sterile water. A 0.1-mL aliquot was mixed with 0.1 mL of 1.5 M Na₂CO₃ containing 4% acetyl acetone and incubated at 100 °C for 20 min, followed by addition of 0.7 mL of 96% ethanol and 0.1 mL of Ehrlich’s reagent (1.6 g p-dimethylaminobenzaldehyde in 30 mL concentrated HCl and 30 mL ethanol). After 1 h at room temperature, absorbance at 520 nm was measured on a SpectraMax M5 plate reader (Molecular Devices). Glucosamine concentrations were derived from a standard curve (0–0.5 mg/mL), and chitin content was expressed as a percentage of cell wall dry weight.

### Broth microdilution

The assay was performed according to the Clinical and Laboratory Standards Institute (CLSI) guidelines (CLSI, 2017) with minor modifications. Briefly, yeast cells in logarithmic phase were harvested, washed twice with sterile distilled water, and resuspended in the same buffer. The cell density was adjusted to 2.5 × 10^3^ cells/mL in YPD broth containing drugs at concentrations ranging from 0.125 to 128 μg/mL. Aliquots of 200 μL of the cell suspension were dispensed into each well of 96-well microtiter plates. Plates were incubated statically at 30 °C for 48 h. Following incubation, cell growth was quantified by measuring the optical density at 600 nm (OD_600_) using a microplate reader. Each condition was tested in triplicate. Control wells contained YPD broth without drugs.

### Whole genome sequencing

DNA extraction, library construction, and sequencing were performed as described previously (Yang et al., 2021a). Data were visualized using Ymap (Abbey et al., 2014). Raw fastq files were uploaded to Ymap (version 2.0).^2^ Read depth was plotted as a function of scaffold position using *C. auris* B11221 genome sequence (Assembly ASM31357562v2, https://www.ncbi.nlm.nih.gov/datasets/genome/GCA_031357565.2/).

### Statistical analysis

All experiments were performed in at least three independent biological replicates, and data are expressed as mean ± standard deviation (SD) unless otherwise indicated. For comparisons between two groups (e.g., parental strain vs. adaptors), unpaired two-tailed Student’s *t*-tests were used. All statistical analyses were conducted using GraphPad Prism 9 (GraphPad Software, San Diego, CA, United States). Exact *p*-values are reported in the Results section or figure legends, and *p* < 0.05 was considered statistically significant.

## Results

### Exposure to tunicamycin selects for resistant survivors

To determine whether exposure to TUN could select for resistant survivors, we first assessed the susceptibility of the parental strain *C auris* B12037 (clade 3) to TUN. Spot assay results showed that growth was visibly inhibited at 2 μg/mL TUN and completely suppressed at concentrations of 4 μg/mL and 8 μg/mL (Figure 1A). Based on these observations, approximately 1 × 10^6^ cells of B12037 were plated onto YPD agar plates containing TUN at concentrations ranging from 2 to 16 μg/mL (Figure 1B). After 5 days of incubation, a limited number of colonies (hereafter referred to as “survivors”) appeared on plates supplemented with 4 μg/mL and 8 μg/mL TUN (Figure 1B). To evaluate whether these survivors had acquired resistance to TUN, we randomly selected 27 colonies from the 4 μg/mL plates and 9 colonies from the 8 μg/mL plates. Spot assays were performed to assess their resistance levels. Among the selected colonies, 10 from the 4 μg/mL selection and 6 from the 8 μg/mL selection exhibited resistance to TUN, as evidenced by their ability to grow at concentrations that fully inhibited the parental strain (Figure 1C). These results indicate that exposure to high doses of TUN selects for a subset of resistant survivors.

**Figure 1:**
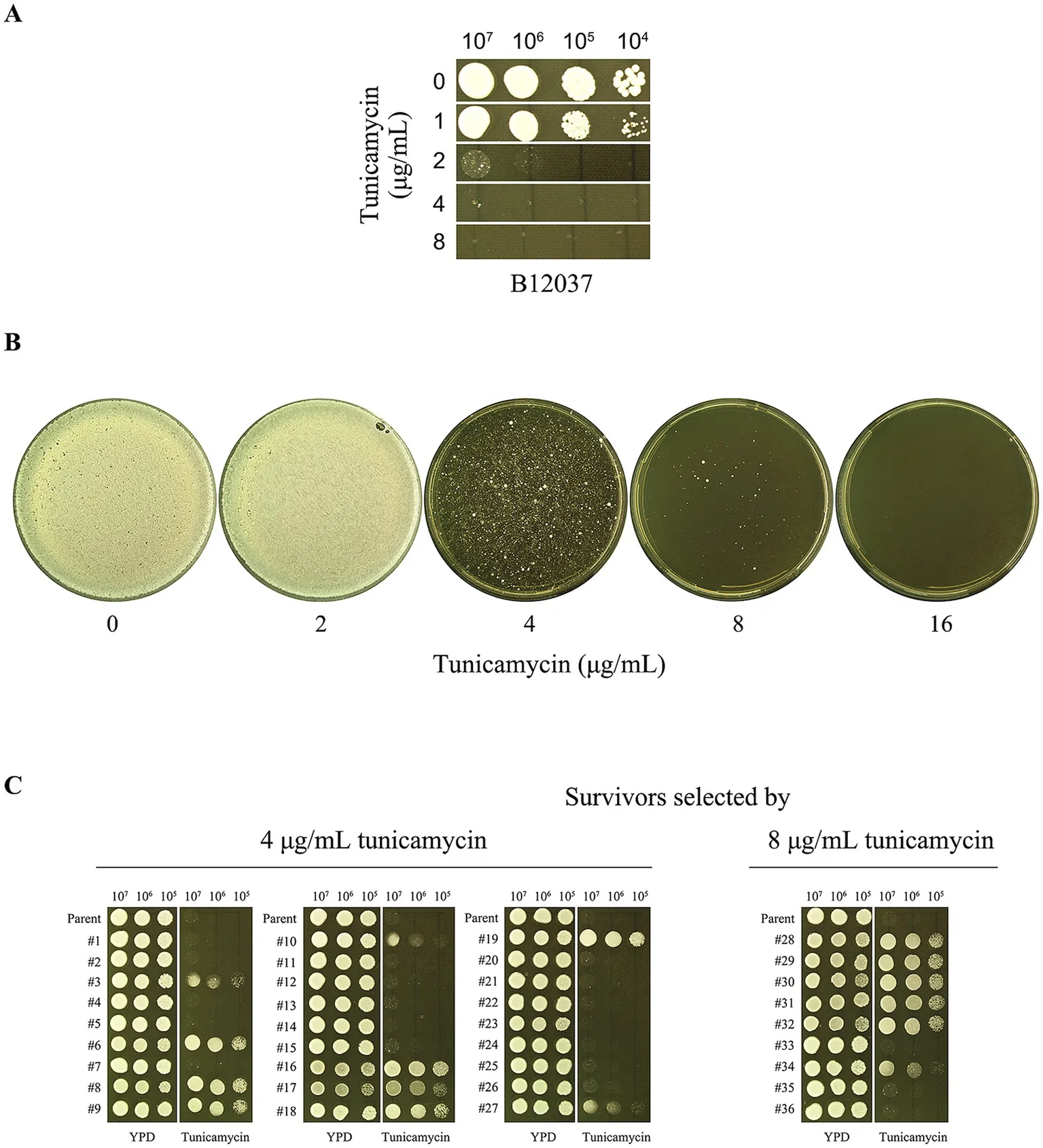
Selection of tunicamycin-resistant survivors in *C. auris.* **(A)** Spot assay of the parental strain B12037 on YPD agar containing increasing concentrations of tunicamycin (0, 2, 4, 8, and 16 μg/mL). Growth was visibly inhibited at 2 μg/mL and completely suppressed at concentrations ≥4 μg/mL. **(B)** Approximately 1 × 10^6^ cells of B12037 were plated onto YPD agar containing 2–16 μg/mL tunicamycin. After 5 days of incubation, a limited number of colonies (survivors) appeared on plates with 4 and 8 μg/mL tunicamycin. From the 4 μg/mL plate, 27 survivors were randomly selected; from the 8 μg/mL plate, 9 survivors were selected for further analysis. **(C)** Spot assays of representative survivors from the 4 μg/mL and 8 μg/mL selection plates compared with the parental strain. Among the selected colonies, 10 from the 4 μg/mL selection and 6 from the 8 μg/mL selection exhibited resistance to tunicamycin, as evidenced by their ability to grow at concentrations that fully inhibited the parental strain.

Similar TUN-induced selection of stress-adapted survivors has been documented in other fungal pathogens. In *C. albicans*, TUN exposure induces chromosome 2 trisomy, which confers cross-tolerance to caspofungin (Yang et al., 2021a), and in *S. cerevisiae*, TUN selection leads to aneuploidy of multiple chromosomes (Beaupere et al., 2018). Our results extend these observations to *C. auris,* demonstrating that TUN can select for survivors with altered antifungal susceptibility in this pathogen.

### Some survivors acquired resistance to fluconazole and/or tolerance to caspofungin

To investigate whether TUN-selected survivors exhibited altered susceptibility to clinically relevant antifungals, we assessed their responses to fluconazole and caspofungin using disk diffusion assays. This method allows discrimination between classical resistance, defined by a reduced or absent zone of inhibition, and tolerance, characterized by visible growth within the inhibition zone despite a clear zone edge.

Among the 16 survivors that displayed resistance to TUN, three isolates (designated #10, #16, and #19) showed markedly altered susceptibility to fluconazole. Specifically, these isolates completely lacked a zone of inhibition around the fluconazole disk, indicating acquired resistance. When tested against caspofungin, two of these three isolates (#10 and #19) distinct colony growth within the zone, indicative of tolerance to caspofungin (Figures 2A,B). In contrast, isolate #16 showed no signs of caspofungin tolerance, displaying a clear zone edge without internal growth.

**Figure 2:**
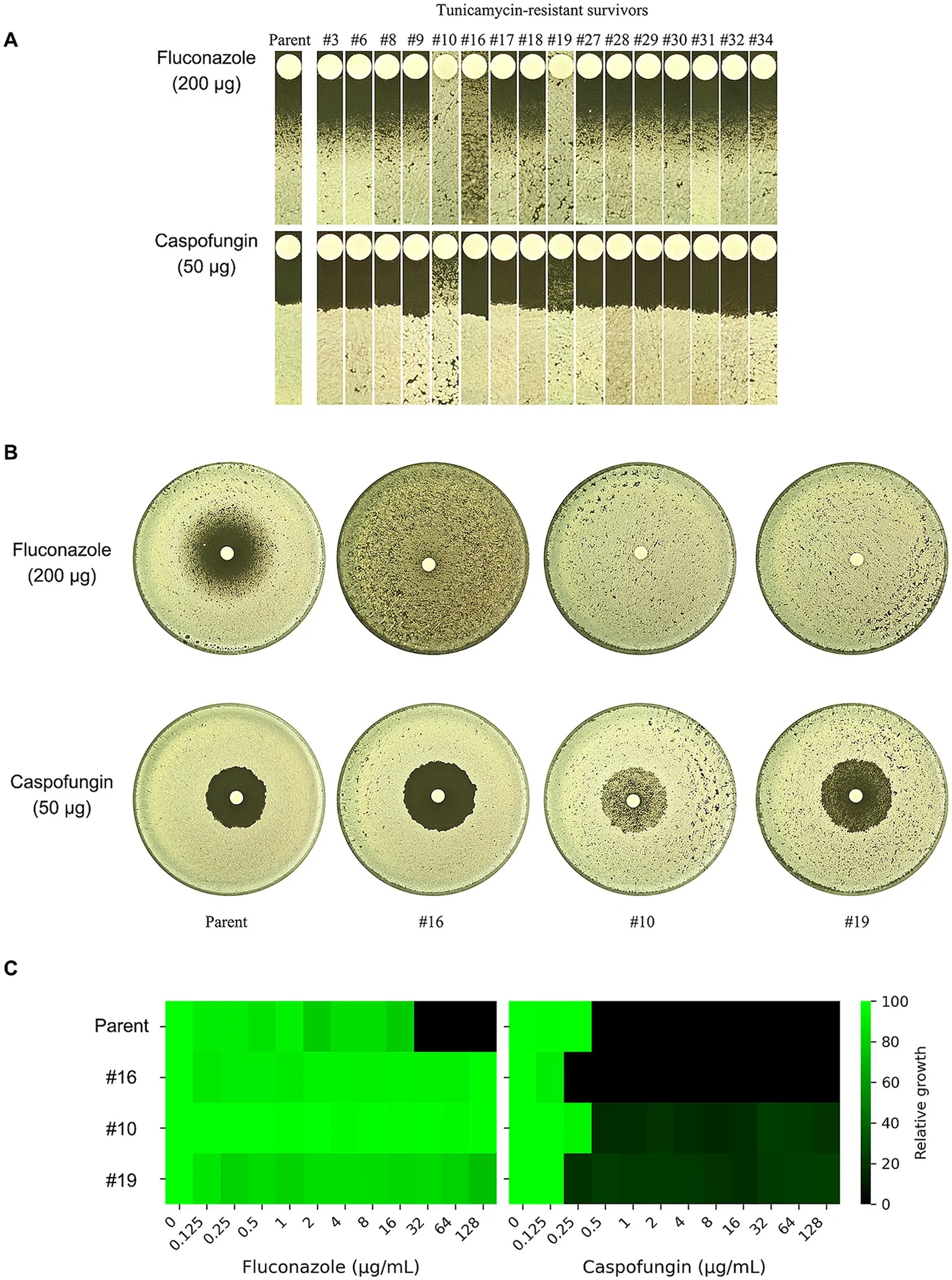
Susceptibility profiles of tunicamycin-selected survivors to fluconazole and caspofungin. **(A)** Disk diffusion assays were performed to evaluate antifungal susceptibility. For fluconazole, the parental strain B12037 showed a clear zone of inhibition. Survivors #10, #16, and #19 displayed no zone of inhibition, indicating acquired resistance to fluconazole. For caspofungin, the parental strain showed a clear zone edge. Survivors #10 and #19 exhibited visible colony growth inside the inhibition zone (foggy growth), indicative of tolerance to caspofungin. Survivor #16 showed a clear zone edge similar to the parental strain, indicating no acquired tolerance. **(B)** Representative full images of parent, #10, #16 and #19 are shown. **(C)** Broth microdilution MIC assays. Minimum inhibitory concentrations (MICs) of fluconazole and caspofungin were determined following CLSI guidelines. For fluconazole, the parental strain showed an MIC of 32 μg/mL, whereas all three survivors exhibited MICs >128 μg/mL, confirming acquired fluconazole resistance. For caspofungin, the parental strain and survivor #16 showed MICs of 0.5 μg/mL and 0.25 μg/mL, respectively, consistent with susceptibility. Although survivors #10 and #19 did not exhibit elevated caspofungin MICs relative to the parental strain, both displayed visible growth (trailing growth) in wells containing drug concentrations above the MIC (≥0.5 μg/mL), indicative of caspofungin tolerance.

To quantitatively validate the antifungal susceptibility profiles observed in disk diffusion assays, we determined the MICs of fluconazole and caspofungin for the parental strain and the three selected surivvors using the broth microdilution method following CLSI guidelines. As shown in Figure 2C, the parental strain B12037 exhibited a fluconazole MIC of 32 μg/mL, whereas all three survivors (#10, #16, and #19) showed MICs >128 μg/mL, confirming their acquired fluconazole resistance. For caspofungin, the parental strain displayed an MIC of 0.5 μg/mL, and isolate #16 exhibited a comparable MIC of 0.25 μg/mL, consistent with its susceptibility to caspofungin as observed in disk diffusion assays. Notably, although isolates #10 and #19 showed no substantial increase in caspofungin MICs relative to the parental strain, both survivors exhibited visible growth in wells containing drug concentrations above the MIC (≥0.5 μg/mL), indicative of caspofungin tolerance. This “trailing growth” phenotype—characterized by reduced but persistent growth at inhibitory concentrations—is a hallmark of antifungal tolerance and is distinct from classical resistance, which would be defined by an elevated MIC.

Collectively, these results demonstrate that exposure to TUN can occasionally select for survivors with enhanced resistance to fluconazole, and in some cases, concomitant tolerance to caspofungin. The distinct susceptibility profiles among the three isolates, together with their divergent transcriptional, functional, and genomic features, indicate that these survivors employ distinct adaptive mechanisms.

The association between caspofungin tolerance and chitin remodeling is consistent with prior reports in *C. auris*. Sharma et al. (2022) showed that clinical isolates lacking *FKS* mutations can exhibit reduced echinocandin susceptibility through increased chitin synthesis, and recent work has demonstrated that stress-induced chitin remodeling contributes to echinocandin survival in this species. Our findings support this model and extend it to TUN-selected survivors.

### Acquired caspofungin tolerance is dependent on Hsp90 and calcineurin

The molecular chaperone Hsp90 and the calcium-activated phosphatase calcineurin are key regulators of fungal stress responses and antifungal tolerance. In *C. albicans*, both Hsp90 and calcineurin have been shown to be required for the acquisition of fluconazole tolerance, and their inhibition can abrogate tolerance without affecting baseline susceptibility in susceptible strains (Cowen and Lindquist, 2005; Singh et al., 2009; Berman and Krysan, 2020). Given their established roles in mediating stress adaptation, we investigated whether these pathways contribute to the caspofungin tolerance observed in the TUN-selected isolates.

To test this, we treated the tolerant isolates #10 and #19 with the Hsp90 inhibitor NVP-HSP990 or the calcineurin inhibitor cyclosporin A (CsA) in combination with caspofungin using disk diffusion assays. In the absence of inhibitors, both isolates exhibited the characteristic “foggy” growth inside the caspofungin inhibition zone, indicative of tolerance. However, upon addition of either NVP-HSP990 or CsA, the growth inside the inhibition zone was abolished, resulting in a clear zone edge similar to that of the susceptible control (Figure 3). This indicates that both Hsp90 and calcineurin are required for the expression of caspofungin tolerance in these isolates.

**Figure 3:**
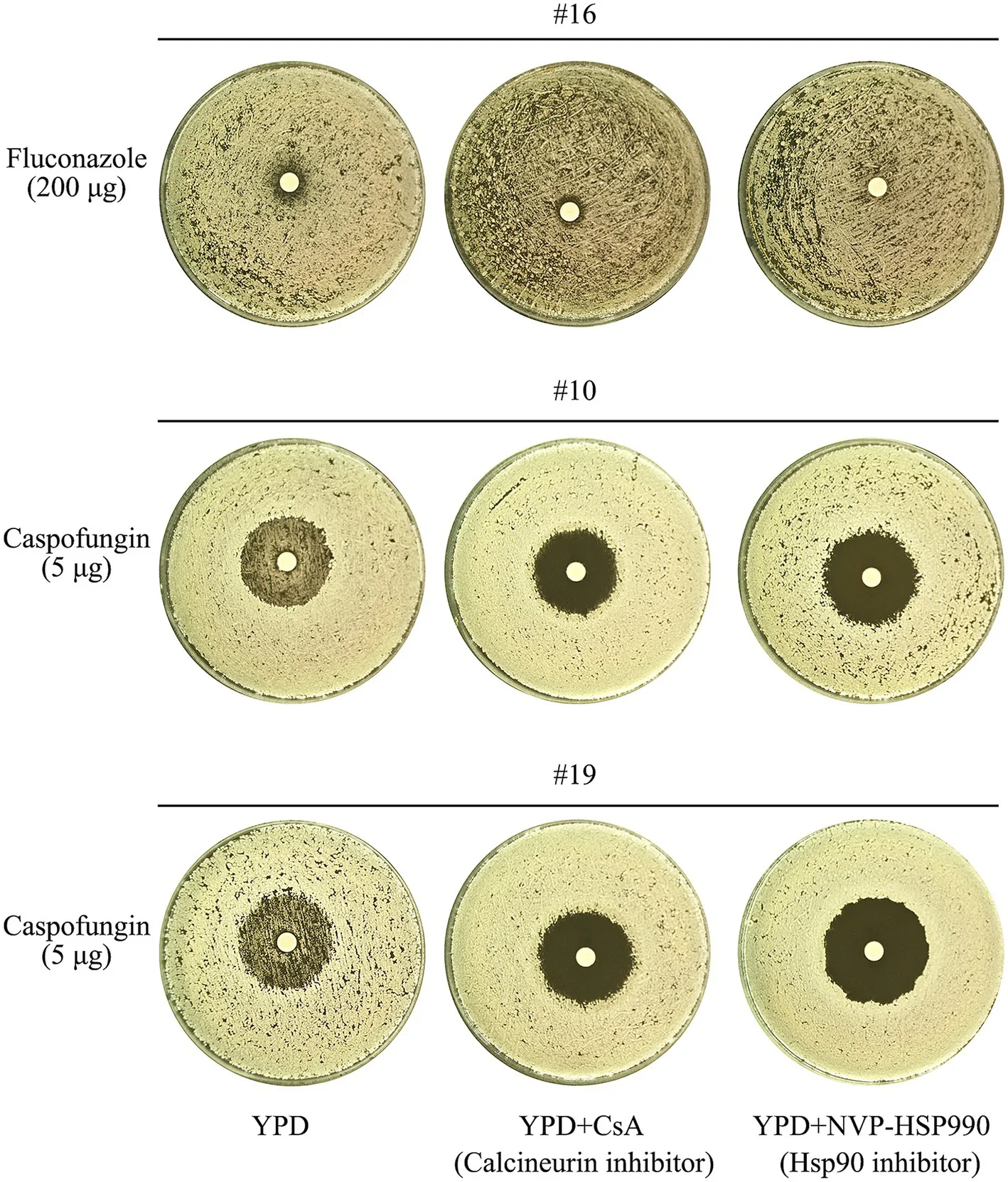
Caspofungin tolerance in tunicamycin-selected survivors depends on Hsp90 and calcineurin. Disk diffusion assays were performed to assess the contribution of Hsp90 and calcineurin to antifungal susceptibility. (Top panel) Fluconazole (200 μg) disks. Survivor #16, which is resistant to fluconazole but susceptible to caspofungin, showed no zone of inhibition in the presence or absence of the Hsp90 inhibitor NVP-HSP990 (1 μg/mL) or the calcineurin inhibitor cyclosporin A (CsA, 0.5 μg/mL), indicating that its fluconazole resistance is independent of these pathways. (Middle and bottom panels) Caspofungin (5 μg) disks. In the absence of inhibitors, survivors #10 and #19 exhibited visible colony growth within the inhibition zone (foggy growth), indicative of tolerance to caspofungin. Addition of either NVP-HSP990 or CsA abolished this intrazonal growth, resulting in a clear zone edge similar to that of the susceptible parental strain. Representative images from three independent experiments are shown.

In contrast, when the fluconazole-resistant but caspofungin-susceptible isolate #16 was tested under the same conditions, neither inhibitor restored fluconazole susceptibility; the zone of inhibition remained absent, consistent with a resistance mechanism that is independent of Hsp90 and calcineurin (Figure 3).

Taken together, these results demonstrate that acquired tolerance to caspofungin in the TUN-selected isolates is dependent on Hsp90 and calcineurin signaling, whereas the fluconazole resistance phenotype in isolate #16 operates through a distinct, Hsp90/calcineurin-independent mechanism. These findings align with the established role of the Hsp90-calcineurin axis in fungal tolerance and highlight the divergent pathways that can emerge under antifungal selection pressure.

### RNA-seq analysis of the transcriptional response to tunicamycin treatment

Having identified that TUN exposure selects for survivors with acquired resistance to fluconazole and tolerance to caspofungin, we next sought to characterize the transcriptional landscape underlying the cellular response to TUN stress. To this end, strain B12037 was treated with 4 μg/mL TUN for 3 h, and the transcriptome was compared with that of an untreated control. Differentially expressed genes were subjected to Gene Ontology (GO) enrichment analysis to identify biological pathways modulated during TUN exposure.

Up-regulated genes were significantly enriched for terms associated with cell wall organization and biogenesis, particularly β-glucan biosynthetic process (odds ratio [OR] = 19.91, *p* = 1.15 × 10^−4^), (1 → 6)-β-D-glucan metabolic process (OR = 33.14, *p* = 2.12 × 10^−4^), and cell wall organization (OR = 11.04, *p* = 1.57 × 10^−3^). These changes indicate reinforcement of the fungal cell wall, which may directly counteract the inhibitory effect of caspofungin, an echinocandin that targets β-glucan synthase. Additionally, transmembrane transport was the most significantly enriched category (OR = 2.2, *p* = 1.02 × 10^−7^), consistent with up-regulation of drug efflux pumps that likely contribute to fluconazole resistance. Other enriched processes included autophagy (OR = 5.81, *p* = 1.67 × 10^−3^) and calcium-mediated signaling (OR = 19.81, *p* = 8.27 × 10^−3^), suggesting activation of stress response and survival pathways under ER stress induced by TUN (Figure 4, left).

**Figure 4:**
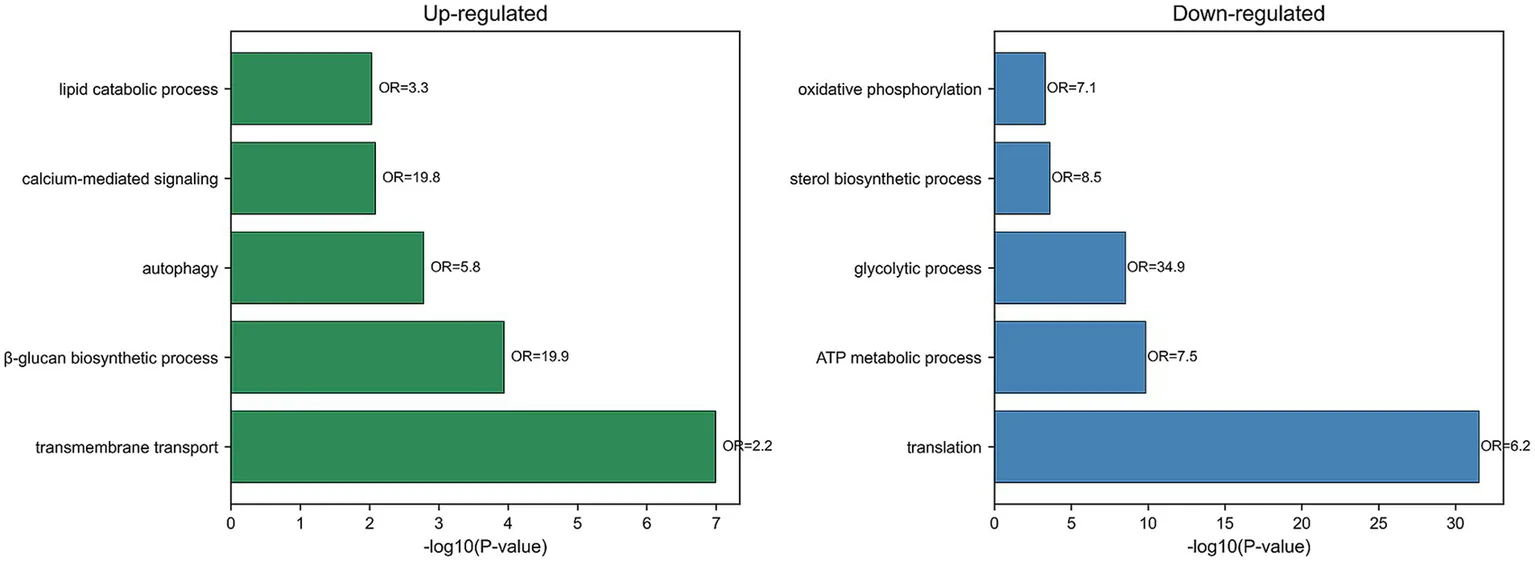
GO enrichment analysis of differentially expressed genes in response to tunicamycin. RNA-seq was performed on the parental strain B12037 treated with 4 μg/mL tunicamycin for 3 h compared to untreated control. Differentially expressed genes were subjected to GO enrichment analysis, and redundant terms sharing parent–child relationships were removed to highlight the most representative biological processes. (Left) Up-regulated genes were significantly enriched for transmembrane transport (OR = 2.2, *p* = 1.02 × 10^−7^), β-glucan biosynthetic process (OR = 19.91, *p* = 1.15 × 10^−4^), autophagy (OR = 5.81, *p* = 1.67 × 10^−3^), calcium-mediated signaling (OR = 19.81, *p* = 8.27 × 10^−3^), and lipid catabolic process (OR = 3.32, *p* = 9.32 × 10^−3^). (Right) Down-regulated genes were enriched for translation (OR = 6.16, *p* = 2.99 × 10^−32^), ATP metabolic process (OR = 7.46, *p* = 1.44 × 10^−10^), sterol biosynthetic process (OR = 8.52, *p* = 2.46 × 10^−4^), oxidative phosphorylation (OR = 7.1, *p* = 4.94 × 10^−4^), and glycolytic process (OR = 34.89, *p* = 3.02 × 10^−9^). Odds ratios (OR) and –log₁₀(*P*-values) are displayed for each term.

Concurrently, genes involved in ATP metabolic process and oxidative phosphorylation were down-regulated, indicating a shift away from mitochondrial respiration. Notably, sterol biosynthetic process was also suppressed, which may alter membrane composition and further affect azole susceptibility (Figure 4, right). Genes involved in translation and peptide biosynthesis were also significantly down-regulated—a transcriptional signature consistent with the global repression of protein synthesis that is a well-established hallmark of the unfolded protein response (UPR) and functions to alleviate ER burden (Wek, 2018; Liu et al., 2024; Gawlak-Socka et al., 2026).

### RNA-seq analysis of resistance-associated genes in survivors

To further characterize the molecular basis of the distinct resistance phenotypes, we performed RNA-seq analysis on the three survivors (#10, #16, and #19) using the wild-type strain as a reference. We focused on genes with established roles in drug efflux, target alterations, and cell wall remodeling (Figure 5).

**Figure 5:**
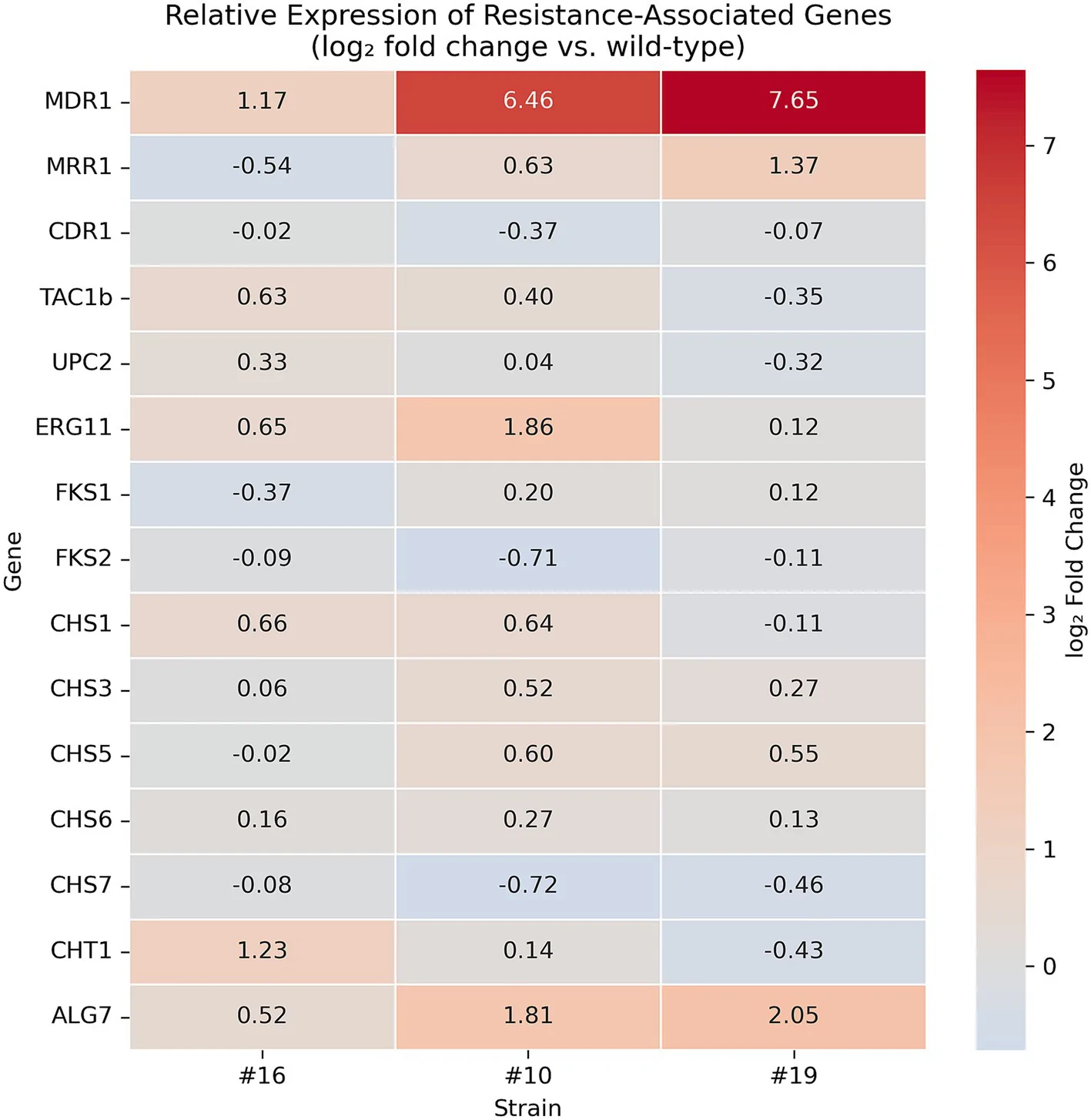
Expression profiles of resistance-associated genes in tunicamycin-selected survivors. RNA-seq analysis was performed on survivors #10, #16, and #19 using the wild-type strain as a reference. Heatmap showing log₂ fold change (log₂FC) values of selected genes involved in drug efflux (*MDR1*, *MRR1*, *CDR1*, *TAC1b*), azole target (*ERG11*), β-glucan synthase (*FKS1*, *FKS2*), chitin synthesis (*CHS1*, *CHS3*, *CHS5*, *CHS6*, *CHS7*), chitin degradation (*CHT1*), and ER stress response (*ALG7*). Red indicates up-regulation; blue indicates down-regulation.

In all three survivors, the efflux pump gene *MDR1* was up-regulated, with the highest expression observed in #10 (log₂FC = 6.46) and #19 (log₂FC = 7.65), followed by #16 (log₂FC = 1.17). The transcription factor *MRR1*, a known regulator of MDR1, showed concordant up-regulation in #19 (log₂FC = 1.37) but was down-regulated in #16 (log₂FC = −0.54) and unchanged in #10 (log₂FC = 0.63). By contrast, *CDR1* expression remained largely unaltered across all strains. The azole target gene *ERG11* was notably up-regulated in #10 (log₂FC = 1.86), moderately increased in #16 (log₂FC = 0.65), and unchanged in #19 (log₂FC = 0.12). These expression patterns are consistent with the observed fluconazole resistance in all three survivors, with #10 displaying a combined mechanism of enhanced efflux and target overexpression.

Expression of the β-glucan synthase catalytic subunit genes *FKS1* and *FKS2* did not show consistent up-regulation in any of the survivors. However, the caspofungin-tolerant survivors #10 and #19 exhibited distinct cell wall remodeling profiles. In #10, several chitin synthase genes were up-regulated, including *CHS1* (log₂FC = 0.64), *CHS3* (log₂FC = 0.52), and *CHS5* (log₂FC = 0.60), suggesting chitin-mediated compensation. In contrast, #16, which remained susceptible to caspofungin, lacked up-regulation of these chitin synthase genes and instead showed up-regulation of the chitinase gene *CHT1* (log₂FC = 1.23). #19 displayed only mild up-regulation of *CHS3* and *CHS5* and down-regulation of *CHT1* (log₂FC = −0.43), indicating that its caspofungin resistance may involve mechanisms beyond chitin remodeling.

The gene *ALG7*, encoding the TUN-sensitive enzyme that catalyzes the first step of N-glycosylation, was up-regulated in all three survivors, with the most pronounced increase observed in #19 (log₂FC = 2.05) and #10 (log₂FC = 1.81), followed by #16 (log₂FC = 0.52). This up-regulation likely reflects a compensatory response to ER stress. Other cell wall integrity-related genes, including several CHS family members and *CHT1*, showed strain-specific expression patterns consistent with their respective caspofungin susceptibility profiles (Figure 5).

In summary, the transcriptomic profiles reveal that fluconazole resistance in all three strains is primarily driven by *MDR1* up-regulation, with additional *ERG11* overexpression contributing in #10. Caspofungin resistance in #10 correlates with chitin synthase up-regulation, while #16 lacks this compensatory response and remains susceptible. The mechanism underlying caspofungin resistance in #19 remains unclear from transcriptional data alone and may involve post-transcriptional or mutational events, possibly reflecting the distinct Hsp90/calcineurin-dependent tolerance pathway we observed.

### Functional validation of efflux activity and chitin content in tunicamycin-selected survivors

To directly corroborate the transcriptomic findings, we performed functional assays to assess efflux pump activity and cell wall chitin content. The efflux of rhodamine 6G (R6G), a fluorescent substrate for multidrug efflux pumps, was measured over time. As shown in Figure 6A, all three fluconazole-resistant survivors (#10, #16, and #19) exhibited significantly higher extracellular R6G accumulation compared to the parental strain at 40, 60, and 80 min (*p* < 0.05, student’s *t*-test), indicating enhanced efflux capacity. This provides direct functional evidence that increased drug efflux, likely mediated by *MDR1* up-regulation, underlies fluconazole resistance.

**Figure 6:**
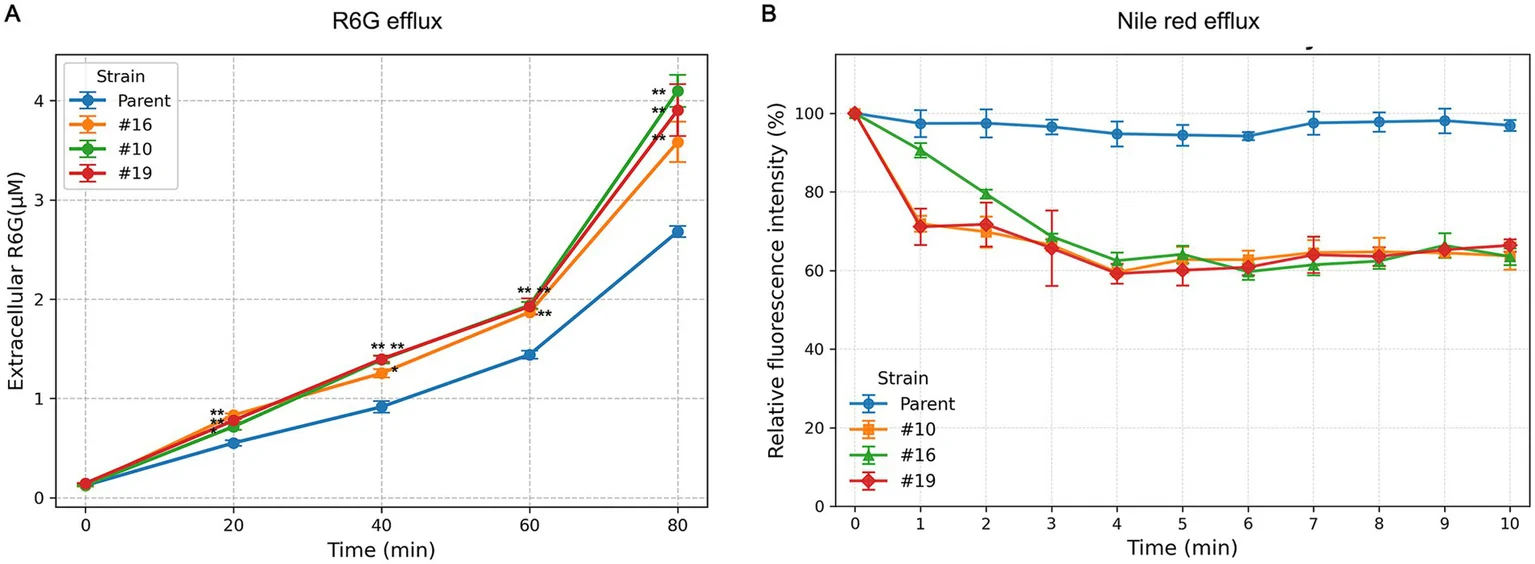
Efflux activity of the parental strain and tunicamycin-selected survivors assessed by R6G and Nile red efflux assays. **(A)** Extracellular rhodamine 6G (R6G) concentration was measured at 0, 20, 40, 60, and 80 min. Data are presented as mean ± SD from three independent biological replicates. **(B)** Nile red efflux was monitored every 1 min for 10 min in the parental strain and the three survivors. Fluorescence at each time point was normalized to the value at time zero. Data are presented as mean ± SD from three independent biological replicates.

Of note, R6G is predominantly transported by ABC-type efflux pumps such as Cdr1 rather than by Mdr1 (Kolaczkowski et al., 1996; Lamping et al., 2007; Niimi et al., 2004), so R6G efflux reflects general efflux capacity rather than Mdr1 activity *per se*. To probe Mdr1-associated efflux more directly, we performed a Nile red efflux assay in parallel, as Nile red has been validated as a substrate of both *C. auris* Mdr1 and Cdr1 in a recombinant *S. cerevisiae* expression system (Toepfer et al., 2023). Nile red efflux was monitored over 10 min in the parental strain and the three survivors, with fluorescence at each time point expressed as a percentage of the initial value at time zero. As shown in Figure 6B, the parental strain retained Nile red throughout the assay, with relative fluorescence intensity remaining close to 100% (96.9 ± 1.4% at 10 min). In contrast, all three survivors showed a progressive and pronounced decline, reaching 63.7 ± 3.5%, 63.5 ± 2.1%, and 66.4 ± 1.6% for #10, #16, and #19, respectively (mean ± SD; *p* < 0.05 versus parental strain, unpaired two-tailed Student’s *t*-test). Thus, Nile red efflux was substantially enhanced in all three survivors, consistent with the up-regulation of *MDR1* detected in these isolates.

In parallel, we quantified cell wall chitin content as a percentage of dry weight. Consistent with the up-regulation of chitin synthase genes in the transcriptomic data, the caspofungin-tolerant isolates #10 and #19 showed significantly elevated chitin levels (8.35 ± 0.05% and 8.08 ± 0.04%, respectively) compared to the parental strain (6.54 ± 0.06%) (Figure 7). In contrast, isolate #16, which is susceptible to caspofungin and lacks chitin synthase up-regulation, showed no significant difference in chitin content (6.42 ± 0.40%). These results confirm that the acquired tolerance to caspofungin is associated with chitin-mediated cell wall remodeling.

**Figure 7:**
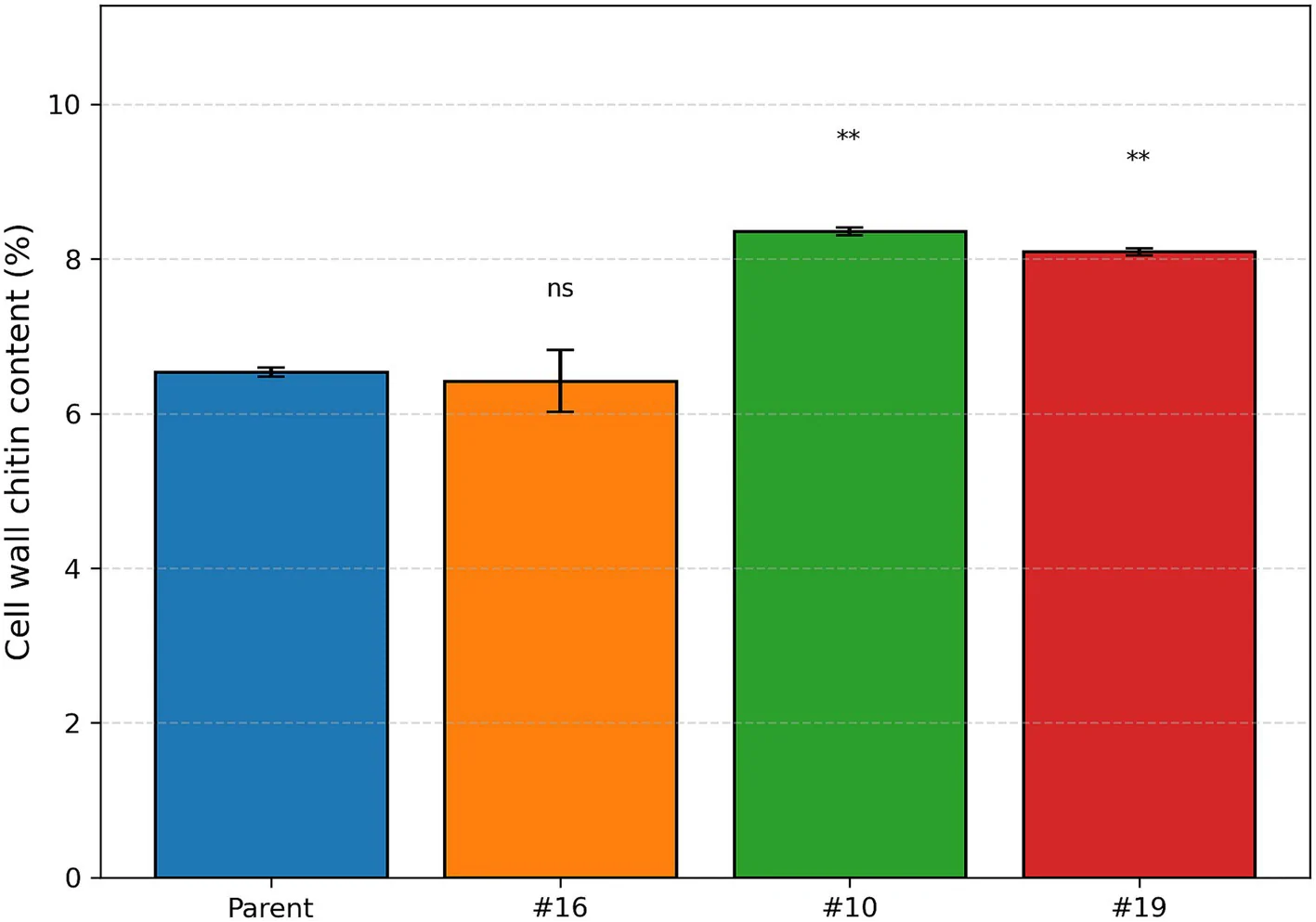
Cell wall chitin content (% dry weight) of the parental strain and survivors. Cell wall chitin content was quantified as a percentage of cell wall dry weight after acid hydrolysis and colorimetric determination of glucosamine. The parental strain exhibited a chitin content of 6.54 ± 0.06%. Survivor #16 (fluconazole-resistant, caspofungin-susceptible) showed no significant difference compared to the parental strain (6.42 ± 0.40%, *p* > 0.05). In contrast, the caspofungin-tolerant survivors #10 and #19 displayed significantly elevated chitin content (8.35 ± 0.05% and 8.08 ± 0.04%, respectively; *p* < 0.05 versus parental strain). Data are presented as mean ± SD from three independent biological replicates. Statistical significance was determined using unpaired two-tailed Student’s *t*-test; **, *p* < 0.001; ns, not significant.

### Whole-genome sequencing reveals segmental aneuploidies in caspofungin-tolerant survivors

To investigate whether chromosomal alterations contribute to the acquired resistance phenotypes, we performed whole-genome sequencing on the parental strain and the three survivors. Ploidy analysis revealed that isolate #16 remained euploid (Figure 8), indicating that its fluconazole resistance is driven primarily by transcriptional changes rather than large-scale genomic rearrangements. In striking contrast, both caspofungin-tolerant isolates #10 and #19 exhibited segmental aneuploidies (Figure 8). Specifically, both isolates carried a segmental disomy of scaffold 5 and 2 to 3 copies of a segmental region on scaffold 6. These karyotypic changes were uniformly present in the sequenced populations, suggesting they were positively selected during TUN exposure.

**Figure 8:**
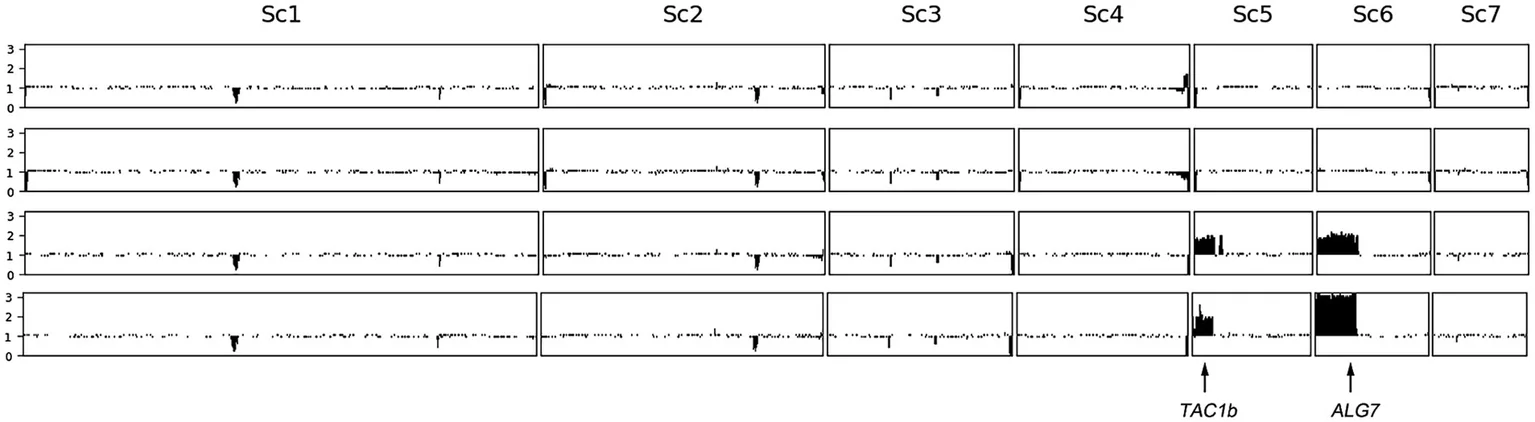
Copy number variation analysis by whole-genome sequencing. Whole-genome sequencing was performed to assess chromosomal copy number variations in the parental strain and tunicamycin-selected survivors. Survivor #16 (fluconazole-resistant, caspofungin-susceptible) showed a normal diploid copy number profile with no detectable segmental aneuploidies. Both caspofungin-tolerant survivors, #10 (B) and #19 (C), exhibited segmental aneuploidies, including a segmental disomy of scaffold 5 and 2–3 copies of a segmental region on scaffold 6. The amplified regions contain *TAC1b* (scaffold 5), a known positive regulator of *MDR1*, and *ALG7* (scaffold 6), which encodes the tunicamycin target enzyme.

We further examined the sequencing reads using Integrative Genomics Viewer (IGV) to screen for point mutations or small indels in canonical antifungal resistance genes, including *TAC1b*, *CDR1*, *MDR1*, *ERG11*, *UPC2*, *FKS1*, *FKS2*, and *CHT1*. No mutations were detected in any of these genes across the three survivors, indicating that the acquired phenotypes are not attributable to sequence alterations in these well-established drug targets or efflux regulators.

Notably, we identified that *TAC1b*—a known positive transcriptional regulator of *MDR1*—is located within the amplified region on scaffold 5, providing a direct genomic link to the robust *MDR1* up-regulation observed in isolates #10 and #19. Additionally, *ALG7*, which encodes the TUN target enzyme (UDP-N-acetylglucosamine—dolichyl-phosphate N-acetylglucosaminephosphotransferase), resides within the amplified region on scaffold 6. The increased copy number of *ALG7* likely represents a direct adaptive response to TUN-induced ER stress by compensating for the drug’s inhibitory effect.

Collectively, these genomic and functional data reveal that ER stress drives the emergence of resistant survivors through two distinct but complementary mechanisms: (i) transcriptional reprogramming in a euploid background (isolate #16), and (ii) segmental aneuploidies that amplify key resistance-associated genes, including *TAC1b* and *ALG7*, in isolates that have acquired both fluconazole resistance and caspofungin tolerance (#10 and #19).

## Discussion

In this study, we investigated the adaptive responses of *C. auris* (clade 3, strain B12037) to TUN, an inducer of endoplasmic reticulum (ER) stress, and characterized the resulting survivors with altered susceptibility to clinically relevant antifungals. Our findings reveal that exposure to TUN selects for survivors that acquire resistance to fluconazole and, in some cases, tolerance to caspofungin. Through transcriptomic and pharmacological analyses, we uncovered distinct molecular mechanisms underlying these phenotypes, highlighting the complex interplay between ER stress adaptation and antifungal drug resistance.

We first demonstrated that TUN treatment, at concentrations that fully inhibit the parental strain, yields a subset of survivors that exhibit resistance to TUN itself. Among these, three survivors (#10, #16, and #19) displayed resistance to fluconazole, while two (#10 and #19) additionally showed tolerance to caspofungin, characterized by growth within the inhibition zone. This phenotypic divergence suggests that the selective pressure imposed by TUN can drive distinct adaptive trajectories. Notably, the observation that not all survivors acquired antifungal resistance underscores that resistance is not an inevitable consequence of TUN exposure but rather arises through specific genetic or epigenetic alterations. The separation of fluconazole resistance and caspofungin tolerance among the isolates further indicates that these phenotypes are governed by independent mechanisms.

Using chemical inhibitors, we found that the Hsp90 inhibitor NVP-HSP990 and the calcineurin inhibitor CsA abrogated caspofungin tolerance in isolates #10 and #19, rendering the inhibition zones clear. In contrast, neither inhibitor affected the fluconazole resistance of isolate #16. These results align with the established role of the Hsp90-calcineurin axis in fungal stress responses, particularly in mediating tolerance to cell wall-active agents such as caspofungin. In *C. albicans* and *Aspergillus fumigatus*, Hsp90 and calcineurin regulate cell wall integrity signaling and are essential for tolerance to echinocandins (Singh et al., 2009; Lamoth et al., 2012). Our data extend this paradigm to *C. auris*, demonstrating that acquired caspofungin tolerance in TUN-selected survivors similarly depends on this conserved stress signaling pathway. The lack of inhibitor effect on fluconazole resistance in #16 suggests that this phenotype operates independently of Hsp90/calcineurin, consistent with the predominant role of efflux pump up-regulation in azole resistance.

RNA-seq analysis of the parental strain exposed to TUN revealed a global transcriptional reprogramming characterized by the up-regulation of cell wall organization and biogenesis, transmembrane transport, and autophagy, alongside down-regulation of translation, oxidative phosphorylation, and sterol biosynthesis. The enrichment of β-glucan biosynthetic processes indicates that cells actively reinforce the cell wall to counteract ER stress, a response that may pre-adapt them to caspofungin challenge. Similarly, the strong enrichment of transmembrane transport genes, including potential drug efflux pumps, likely contributes to the observed fluconazole resistance. The concomitant activation of autophagy and calcium-mediated signaling reflects a broader survival strategy to manage ER stress. The global repression of translation and energy metabolism is a hallmark of the unfolded protein response, which alleviates ER burden by reducing protein synthesis (Wek, 2018; Liu et al., 2024) and reallocating resources toward stress-adaptive pathways (Kim et al., 2024; Gawlak-Socka et al., 2026). The suppression of sterol biosynthesis may alter membrane composition, potentially affecting azole susceptibility (Elsaman et al., 2024; Li et al., 2024). Collectively, these data paint a picture of a finely tuned cellular response that prioritizes stress survival over growth, creating a permissive state for the emergence of antifungal resistance.

Analysis of the three survivors revealed distinct transcriptional signatures that correlate with their resistance phenotypes. In all survivors, *MDR1* was up-regulated, supporting its central role in fluconazole resistance. Notably, isolate #10 also exhibited up-regulation of *ERG11*, the azole target, suggesting a dual mechanism of resistance combining enhanced efflux with target overexpression. Such combinatorial strategies have been described in clinical isolates of *C. auris* and are associated with higher-level resistance (Toepfer et al., 2023; Barker et al., 2026). The lack of *CDR1* up-regulation further underscores the predominant contribution of the *MDR1* efflux system in this strain background.

Regarding caspofungin tolerance, isolate #10 showed up-regulation of multiple chitin synthase genes (*CHS1*, *CHS3*, *CHS5*), consistent with a compensatory cell wall remodeling mechanism frequently observed in echinocandin-tolerant or -resistant isolates. In contrast, isolate #16, which remains caspofungin susceptible, lacked chitin synthase up-regulation but exhibited increased expression of the chitinase gene *CHT1*, which may attenuate cell wall chitin content and thereby maintain sensitivity. Isolate #19 displayed a more subtle pattern, with only modest changes in chitin synthase expression and down-regulation of *CHT1*, suggesting that its caspofungin tolerance may involve alternative mechanisms, possibly including mutations in FKS genes or post-transcriptional regulation. The universal up-regulation of *ALG7*, the target of TUN (Beaupere et al., 2018), confirms that all survivors retain a compensatory response to ER stress, even after selection.

The functional validation of efflux activity and chitin content further strengthens the mechanistic conclusions drawn from the transcriptomic data. The Nile red efflux data provide direct functional evidence for Mdr1-associated efflux in the TUN-selected survivors. Unlike R6G, which is primarily an ABC-transporter substrate, Nile red has been validated as a substrate of *C. auris* Mdr1 and Cdr1 (Toepfer et al., 2023). The concordance between *MDR1* up-regulation and enhanced Nile red efflux across all three survivors supports a role for Mdr1 in the fluconazole resistance phenotype, and resolves the apparent inconsistency between the R6G results and the absence of *CDR1* up-regulation.

Meanwhile, the elevated chitin content specifically in the caspofungin-tolerant isolates provides biochemical evidence for the chitin-mediated cell wall reinforcement pathway. These functional assays not only validate our RNA-seq findings but also underscore the distinct adaptive strategies—efflux pump activation versus cell wall remodeling—that *C. auris* employs to survive ER stress and acquire antifungal resistance.

The WGS data revealed a striking dichotomy in the adaptive strategies employed by the TUN-selected survivorss. Isolate #16, which acquired fluconazole resistance but remained caspofungin-susceptible, maintained a strictly euploid genome, indicating that fluconazole resistance can be achieved through transcriptional reprogramming alone (*MDR1* up-regulation) without large-scale genomic rearrangements. In contrast, both caspofungin-tolerant isolates (#10 and #19) harbored segmental aneuploidies—specifically, segmental disomy of scaffold 5 and 2–3 copies of a segmental region on scaffold 6. Unlike whole-chromosome aneuploidy documented in ER stress-induced resistance in other fungi (Beaupere et al., 2018; Yang et al., 2021a,b; Zheng et al., 2024a), the segmental nature of these alterations suggests a more refined adaptive strategy that may minimize fitness costs while providing gene dosage benefits.

Segmental aneuploidies in our *C. auris* survivors are consistent with the growing recognition that stress-induced genomic plasticity contributes to antifungal resistance across pathogenic fungi. In *C. albicans*, TUN exposure selects chromosome 2 trisomy that confers caspofungin cross-tolerance (Yang et al., 2021a,b), and in *C. neoformans*, brefeldin A induces aneuploidy conferring fluconazole resistance (Zhang et al., 2024). In *C. glabrata*, TUN drives both petite formation and chromosome C aneuploidy (Dong et al., 2025). Our finding of segmental, rather than whole-chromosome, aneuploidies in *C. auris* suggests a more refined adaptive strategy that may minimize fitness costs while providing gene dosage benefits.

Importantly, IGV analysis confirmed the absence of point mutations in all canonical resistance genes (*TAC1b*, *CDR1*, *MDR1*, *ERG11*, *UPC2*, *FKS1*, *CHT1*), indicating that the acquired phenotypes are driven by transcriptional changes rather than classical target-site alterations.

The contribution of *TAC1b* to antifungal resistance in *C. auris* has been predominantly attributed to gain-of-function (GOF) mutations that drive *CDR1* overexpression and, to a lesser extent, *MDR1* up-regulation (Rybak et al., 2020; Barker et al., 2026). In our study, no *TAC1b* GOF mutations were detected in the three TUN-selected survivors. Moreover, *TAC1b* (CJI97_004632; 0.189540–0.192140 Mb) is not located within the amplified regions on scaffold 5 in isolates #10 and #19. The amplification in #10 spans the left telomere to 0.172 Mb and 0.215–0.240 Mb, while in #19 it extends from the left telomere to 0.172 Mb; neither region encompasses *TAC1b*. These observations distinguish our survivors from the canonical *TAC1b*-mediated resistance paradigm. In contrast to *TAC1b* GOF mutants, which typically exhibit *CDR1* up-regulation (Barker et al., 2026), our survivors showed no *CDR1* induction despite robust *MDR1* up-regulation. *TAC1b*-independent routes to azole resistance have been reported: Mayr et al. (2020) showed that Tac1b contributes to azole resistance through a *CDR1*-independent mechanism, and Carolus et al. (2021) reported that chromosome 5 duplication increased *ERG11*, *TAC1b*, and *CDR2* expression but not *CDR1*. In addition, the Mrr1–Mdr1 axis can mediate azole resistance independently of *CDR1* (Li et al., 2022). Our findings are consistent with these reports and suggest that fluconazole resistance in these survivors operates through a *TAC1b*-independent pathway, with Mdr1-mediated efflux as the primary driver.

Collectively, these findings demonstrate that *C. auris* can employ two fundamentally distinct adaptive strategies in response to ER stress: purely transcriptional adaptation in a euploid background (isolate #16) versus combined transcriptional and genomic rearrangements via segmental aneuploidy (isolates #10 and #19). The association of segmental aneuploidy with the more complex multidrug-resistant phenotype raises the possibility that such genomic alterations may facilitate the evolution of pan-resistance in this highly plastic pathogen. Future studies employing long-read sequencing and functional genetic validation will be required to definitively establish the causal role of these segmental aneuploidies.

Our findings are consistent with a growing body of literature demonstrating that ER stress can drive the emergence of antifungal resistance and tolerance through both transcriptional and genomic mechanisms. In *Cryptococcus neoformans*, TUN induced ER stress has been shown to promote aneuploidy formation, with some aneuploid strains conferring resistance or cross-resistance to fluconazole and 5-flucytosine (Zheng et al., 2024a). Similarly, in *C. albicans*, exposure to azoles such as miconazole and ketoconazole induces aneuploidy-mediated tolerance that is dependent on Hsp90 and calcineurin (Guo et al., 2024; Zheng et al., 2024b), and caspofungin paradoxical growth has been linked to segmental aneuploidy on Chromosome R that drives broad transcriptional remodeling (Wei et al., 2025). The segmental aneuploidies we observed in *C. auris*—specifically on scaffolds 5 and 6, harboring TAC1b and ALG7—extend these observations to a new pathogen and suggest that stress-induced genomic plasticity may be a conserved adaptive strategy among pathogenic fungi. The requirement of Hsp90 and calcineurin for caspofungin tolerance in the survivors further aligns with the established role of these pathways in mediating stress adaptation and drug tolerance.

Collectively, our results demonstrate that exposure to ER stress can drive the emergence of survivors with multidrug resistance traits in *C. auris*, a pathogen already notorious for its propensity to develop antifungal resistance. The mechanistic dissection presented here highlights two distinct pathways: a Collectively, our results demonstrate that exposure to ER stress can drive the emergence of survivors with multidrug resistance traits in *C. auris*, a pathogen already notorious for its propensity to develop antifungal resistance. The mechanistic dissection presented here highlights two distinct pathways: an Hsp90/calcineurin-dependent tolerance to caspofungin associated with chitin remodeling, and an Hsp90/calcineurin-independent fluconazole resistance associated with *MDR1* up-regulation and enhanced Mdr1-mediated efflux.

Several limitations of this study should be acknowledged. First, our experiments were conducted using a single *C. auris* isolate from clade III (African clade). *C. auris* comprises multiple genetically distinct clades (I–VI) that differ in geographic distribution, antifungal susceptibility profiles, and resistance mechanisms (Gifford et al., 2024). Clades I, III, and IV are associated with multidrug resistance, whereas clades II and VI exhibit lower resistance rates (da Silva et al., 2025). Clade-associated point mutations in *ERG11*—including F126L in clade III—have been linked to azole resistance, underscoring the genetic heterogeneity across clades (Kappel et al., 2024; Barker et al., 2026). Whether the ER stress-driven selection of fluconazole-resistant and caspofungin-tolerant survivors via *MDR1* up-regulation and segmental aneuploidy observed in clade III is generalizable to other clades remains to be determined. The specific genomic regions subject to amplification (e.g., scaffolds 5 and 6 harboring *TAC1b* and *ALG7*) and the precise transcriptional responses may differ due to variations in genome architecture, regulatory networks, and baseline stress responses across clades. Future studies employing multiple isolates from clades I, II, IV, V, and VI will be essential to establish the breadth of these adaptive mechanisms across the *C. auris* species complex.

## Conclusion

In summary, this study reveals that TUN-induced ER stress selects for *C. auris* survivors with acquired resistance to fluconazole and tolerance to caspofungin. These phenotypes arise through distinct molecular mechanisms: fluconazole resistance is associated with *MDR1* up-regulation and enhanced Mdr1-associated efflux, whereas caspofungin tolerance depends on the Hsp90-calcineurin axis and chitin remodeling. Our findings underscore the capacity of *C. auris* to adapt to stress in ways that confer cross-resistance to clinically important antifungals, highlighting the need for therapeutic strategies that target adaptive pathways to prevent resistance emergence.

## Data Availability

The datasets presented in this study can be found in online repositories. The names of the repository/repositories and accession number(s) can be found at: https://www.ebi.ac.uk/arrayexpress/, E-MTAB-16877.

## Footnotes

1
https://www.candidagenome.org/download/sequence/C_auris_B11221/current/

2
http://cse-helix24.cs.umn.edu/ymap/

## References

[ref1] Abbey D. A. Funt J. Lurie-Weinberger M. N. Thompson D. A. Regev A. Myers C. L. . (2014). YMAP: a pipeline for visualization of copy number variation and loss of heterozygosity in eukaryotic pathogens. Genome Med. 6:100. doi: 10.1186/s13073-014-0100-8, 25505934 PMC4263066

[ref2] Athavale-Wad J. Gopalakrishnan R. Krishna V. Sethuraman N. Nambi P. S. Sridharan S. . (2025). Comparative clinical characteristics and outcomes of *Candida (Candidozyma) auris* vs. non-*C. auris* candidemia in non-neutropenic patients in South India. Mycopathologia 190:72. doi: 10.1007/s11046-025-00974-2 40815478

[ref3] Barker K. S. Santana D. J. Zhang Q. Peters T. L. Rybak J. M. Morschhauser J. . (2025). Mutations in TAC1B drive increased CDR1 and MDR1 expression and azole resistance in *Candida auris*. Antimicrob. Agents Chemother. 69:e0030025. doi: 10.1128/aac.00300-25, 40824653 PMC12486835

[ref4] Barker K. S. Zhang Q. Peters T. L. Rybak J. M. Morschhauser J. Cuomo C. A. . (2026). Relative contributions of the ERG11(VF125AL) and MRR1A(N647T) mutations to fluconazole resistance in clade III *Candidozyma (Candida) auris* clinical isolates. Clin. Microbiol. Infect. 32, 285–291. doi: 10.1016/j.cmi.2025.10.009, 41115594 PMC12713649

[ref5] Beaupere C. Dinatto L. Wasko B. M. Chen R. B. VanValkenburg L. Kiflezghi M. G. . (2018). Genetic screen identifies adaptive aneuploidy as a key mediator of ER stress resistance in yeast. Proc. Natl. Acad. Sci. USA 115, 9586–9591. doi: 10.1073/pnas.1804264115, 30185560 PMC6156608

[ref6] Berman J. Krysan D. J. (2020). Drug resistance and tolerance in fungi. Nat. Rev. Microbiol. 18, 319–331. doi: 10.1038/s41579-019-0322-2, 32047294 PMC7231573

[ref7] Carolus H. Pierson S. Munoz J. F. Subotic A. Cruz R. B. Cuomo C. A. . (2021). Genome-wide analysis of experimentally evolved *Candida auris* reveals multiple novel mechanisms of multidrug resistance. mBio 12. doi: 10.1128/mBio.03333-20 PMC8092288 33820824

[ref8] Centers for Disease Control and Prevention (2019). Antibiotic Resistance Threats in the United States, 2019. Atlanta: U.S. Department of Health and Human Services, Centers for Disease Control and Prevention.

[ref9] Centers for Disease Control and Prevention (2025). Clinical Treatment of *C. auris* Infections. Atlanta, GA: Centers for Disease Control and Prevention (US).

[ref10] Clinical and Laboratory Standards Institute (2009). M44-A2: Method for Antifungal Disk Diffusion Susceptibility Testing of Yeasts; Approved Guideline—Second Edition. Wayne: Clinical and Laboratory Standards Institute.

[ref11] CLSI (2017). "Reference Method for Broth Dilution Antifungal Susceptibility Testing of Yeasts. 4th ed. CLSI standard M27". Wayne: Clinical and Laboratory Standards Institute.

[ref12] Coste A. T. Karababa M. Ischer F. Bille J. Sanglard D. (2004). TAC1, transcriptional activator of CDR genes, is a new transcription factor involved in the regulation of *Candida albicans* ABC transporters CDR1 and CDR2. Eukaryot. Cell 3, 1639–1652. doi: 10.1128/EC.3.6.1639-1652.2004, 15590837 PMC539021

[ref13] Cowen L. E. Lindquist S. (2005). Hsp90 potentiates the rapid evolution of new traits: drug resistance in diverse fungi. Science 309, 2185–2189. doi: 10.1126/science.1118370, 16195452

[ref14] da Silva K. J. G. Lucini F. Dos Santos R. A. C. Santos D. A. Meis J. F. Melhem M. S. C. . (2025). How does antifungal resistance vary in *Candida (Candidozyma) auris* and its clades? Quantitative and qualitative analyses and their clinical implications. Clin. Microbiol. Infect. 31, 1146–1156. doi: 10.1016/j.cmi.2025.04.003 40216246

[ref15] Dong Y. Ma C. Wang J. Bai S. Wang C. Xu Y. (2025). Dual adaptive strategies in Candida glabrata under tunicamycin stress: petite mutations and chromosome C aneuploidy drive transient drug resistance. Front. Microbiol. 16:1675175. doi: 10.3389/fmicb.2025.1675175, 41195397 PMC12583059

[ref16] Doorley L. A. Meza-Perez V. Jones S. J. Rybak J. M. (2025). A Candidozyma (Candida) auris-optimized episomal plasmid-induced Cas9-editing system reveals the direct impact of the S639F-encoding FKS1 mutation. J. Infect. Dis. 232, e529–e536. doi: 10.1093/infdis/jiaf285, 40569759 PMC12455316

[ref17] Eix E. F. Nett J. E. (2025). Candida auris: epidemiology and antifungal strategy. Annu. Rev. Med. 76, 57–67. doi: 10.1146/annurev-med-061523-021233, 39656947 PMC11808652

[ref18] Elsaman H. Golubtsov E. Brazil S. Ng N. Klugherz I. Martin R. . (2024). Toxic eburicol accumulation drives the antifungal activity of azoles against *Aspergillus fumigatus*. Nat. Commun. 15:6312. doi: 10.1038/s41467-024-50609-1, 39060235 PMC11282106

[ref19] Gawlak-Socka S. Kowalczyk E. Wiktorowska-Owczarek A. (2026). Unfolded protein response at the crossroads: integrating endoplasmic reticulum stress with cellular stress networks. Int. J. Mol. Sci. 27. doi: 10.3390/ijms27041986, 41752123 PMC12940255

[ref20] Gerstein A. C. Rosenberg A. Hecht I. Berman J. (2016). diskImageR: quantification of resistance and tolerance to antimicrobial drugs using disk diffusion assays. Microbiology (Reading) 162, 1059–1068. doi: 10.1099/mic.0.000295, 27126388 PMC5756480

[ref21] Gifford H. Rhodes J. Farrer R. A. (2024). The diverse genomes of Candida auris. Lancet Microbe 5:100903. doi: 10.1016/S2666-5247(24)00135-6 38889739

[ref22] Guo L. Zheng L. Dong Y. Wang C. Deng H. Wang Z. . (2024). Miconazole induces aneuploidy-mediated tolerance in *Candida albicans* that is dependent on Hsp90 and calcineurin. Front. Cell. Infect. Microbiol. 14:1392564. doi: 10.3389/fcimb.2024.1392564, 38983116 PMC11231705

[ref23] Kappel D. Gifford H. Brackin A. Abdolrasouli A. Eyre D. W. Jeffery K. . (2024). Genomic epidemiology describes introduction and outbreaks of antifungal drug-resistant Candida auris. NPJ Antimicrob. Resist. 2:26. doi: 10.1038/s44259-024-00043-6, 39359891 PMC11442302

[ref24] Kim H. Chen Q. Ju D. Purandare N. Chen X. Samavati L. . (2024). ER-tethered stress sensor CREBH regulates mitochondrial unfolded protein response to maintain energy homeostasis. Proc. Natl. Acad. Sci. USA 121:e2410486121. doi: 10.1073/pnas.2410486121, 39589874 PMC11626163

[ref9001] Kolaczkowski M. van der Rest M. Cybularz-Kolaczkowska A. Soumillion J. P. Konings W. N. Goffeau A. (1996). Anticancer drugs, ionophoric peptides, and steroids as substrates of the yeast multidrug transporter Pdr5p. J Biol Chem. 271, 31543–31548. doi: 10.1074/jbc.271.49.31543, 8940170

[ref25] Kukurudz R. J. Chapel M. Wonitowy Q. Adamu Bukari A. R. Sidney B. Sierhuis R. . (2022). Acquisition of cross-azole tolerance and aneuploidy in *Candida albicans* strains evolved to posaconazole. G3 12. doi: 10.1093/g3journal/jkac156, 35881695 PMC9434289

[ref26] Lamoth F. Juvvadi P. R. Fortwendel J. R. Steinbach W. J. (2012). Heat shock protein 90 is required for conidiation and cell wall integrity in *Aspergillus fumigatus*. Eukaryot. Cell 11, 1324–1332. doi: 10.1128/EC.00032-12, 22822234 PMC3486032

[ref9002] Lamping E. Monk B. C. Niimi K. Holmes A. R. Tsao S. Tanabe K. (2007). Characterization of three classes of membrane proteins involved in fungal azole resistance by functional hyperexpression in Saccharomyces cerevisiae. Eukaryot Cell. 6, 1150–1165. doi: 10.1128/EC.00091-07, 17513564 PMC1951111

[ref27] Li J. Aubry L. Brandalise D. Coste A. T. Sanglard D. Lamoth F. (2024). Upc2-mediated mechanisms of azole resistance in *Candida auris*. Microbiol. Spectrum 12:e0352623. doi: 10.1128/spectrum.03526-23, 38206035 PMC10845950

[ref28] Li J. Coste A. T. Bachmann D. Sanglard D. Lamoth F. (2022). Deciphering the Mrr1/Mdr1 pathway in azole resistance of Candida auris. Antimicrob. Agents Chemother. 66:e0006722. doi: 10.1128/aac.00067-22, 35343781 PMC9017311

[ref29] Liu Y. Gu Y. Chen Y. Wang X. Zhou G. Li J. . (2024). Translocational attenuation mediated by the PERK-SRP14 axis is a protective mechanism of unfolded protein response. Cell Rep. 43:114402. doi: 10.1016/j.celrep.2024.114402, 38943644

[ref30] Love M. I. Huber W. Anders S. (2014). Moderated estimation of fold change and dispersion for RNA-seq data with DESeq2. Genome Biol. 15:550. doi: 10.1186/s13059-014-0550-8, 25516281 PMC4302049

[ref31] Marichal P. Koymans L. Willemsens S. Bellens D. Verhasselt P. Luyten W. . (1999). Contribution of mutations in the cytochrome P450 14alpha-demethylase (Erg11p, Cyp51p) to azole resistance in *Candida albicans*. Microbiology (Reading) 145, 2701–2713. doi: 10.1099/00221287-145-10-2701 10537192

[ref32] Massic L. Doorley L. A. Jones S. J. Richardson I. Siao D. D. Siao L. . (2025). Acquired amphotericin B resistance attributed to a mutated ERG3 in Candidozyma auris. Antimicrob. Agents Chemother. 69:e0060125. doi: 10.1128/aac.00601-25, 40980913 PMC12587534

[ref33] Mayr E. M. Ramirez-Zavala B. Kruger I. Morschhauser J. (2020). A zinc cluster transcription factor contributes to the intrinsic fluconazole resistance of *Candida auris*. mSphere 5. doi: 10.1128/mSphere.00279-20, 32321822 PMC7178551

[ref34] Misas E. Parnell L. A. Rajeev M. Lopez L. F. Santos A. Mudge Z. B. . (2025). A benchmark dataset for validating *FKS1* mutations in *Candida auris*. Microbiol. Spectrum 13:e0314724. doi: 10.1128/spectrum.03147-24, 40530813 PMC12323592

[ref9003] Niimi M. Niimi K. Takano Y. Holmes A. R. Fischer F. J. Uehara Y. . (2004). Regulated overexpression of CDR1 in Candida albicans confers multidrug resistance. J Antimicrob Chemother, 54, 999–1006. doi: 10.1093/jac/dkh456, 15486081

[ref35] Rasouli Koohi S. Shankarnarayan S. A. Galon C. M. Charlebois D. A. (2023). Identification and elimination of antifungal tolerance in Candida auris. Biomedicine 11. doi: 10.3390/biomedicines11030898, 36979876 PMC10045952

[ref36] Richie D. L. Hartl L. Aimanianda V. Winters M. S. Fuller K. K. Miley M. D. . (2009). A role for the unfolded protein response (UPR) in virulence and antifungal susceptibility in *Aspergillus fumigatus*. PLoS Pathog. 5:e1000258. doi: 10.1371/journal.ppat.1000258, 19132084 PMC2606855

[ref37] Rosenberg A. Ene I. V. Bibi M. Zakin S. Segal E. S. Ziv N. . (2018). Antifungal tolerance is a subpopulation effect distinct from resistance and is associated with persistent candidemia. Nat. Commun. 9:2470. doi: 10.1038/s41467-018-04926-x, 29941885 PMC6018213

[ref38] Rybak J. M. Munoz J. F. Barker K. S. Parker J. E. Esquivel B. D. Berkow E. L. . (2020). Mutations in *TAC1B*: a novel genetic determinant of clinical fluconazole resistance in *Candida auris*. MBio 11. doi: 10.1128/mBio.00365-20, 32398311 PMC7218281

[ref39] Satoh K. Makimura K. Hasumi Y. Nishiyama Y. Uchida K. Yamaguchi H. (2009). *Candida auris* sp. nov., a novel ascomycetous yeast isolated from the external ear canal of an inpatient in a Japanese hospital. Microbiol. Immunol. 53, 41–44. doi: 10.1111/j.1348-0421.2008.00083.x 19161556

[ref40] Sharma D. Paul R. A. Rudramurthy S. M. Kashyap N. Bhattacharya S. Soman R. . (2022). Impact of FKS1 genotype on echinocandin in vitro susceptibility in Candida auris and in vivo response in a murine model of infection. Antimicrob. Agents Chemother. 66:e0165221. doi: 10.1128/AAC.01652-21, 34780273 PMC8765266

[ref41] Singh S. D. Robbins N. Zaas A. K. Schell W. A. Perfect J. R. Cowen L. E. (2009). Hsp90 governs echinocandin resistance in the pathogenic yeast *Candida albicans* via calcineurin. PLoS Pathog. 5:e1000532. doi: 10.1371/journal.ppat.1000532, 19649312 PMC2712069

[ref42] Sun L. L. Li H. Yan T. H. Cao Y. B. Jiang Y. Y. Yang F. (2023a). Aneuploidy enables cross-tolerance to unrelated antifungal drugs in *Candida parapsilosis*. Front. Microbiol. 14:1137083. doi: 10.3389/fmicb.2023.1137083, 37113223 PMC10126355

[ref43] Sun L. L. Li H. Yan T. H. Fang T. Wu H. Cao Y. B. . (2023b). Aneuploidy mediates rapid adaptation to a subinhibitory amount of fluconazole in *Candida albicans*. Microbiol. Spectrum 11:e0301622. doi: 10.1128/spectrum.03016-22, 36853047 PMC10101127

[ref44] Todd R. T. Soisangwan N. Peters S. Kemp B. Crooks T. Gerstein A. . (2023). Antifungal drug concentration impacts the Spectrum of adaptive mutations in *Candida albicans*. Mol. Biol. Evol. 40. doi: 10.1093/molbev/msad009, 36649220 PMC9887641

[ref45] Toepfer S. Lackner M. Keniya M. V. Monk B. C. (2023). Functional expression of recombinant *Candida auris* proteins in *Saccharomyces cerevisiae* enables azole susceptibility evaluation and drug discovery. J. Fungi 9. doi: 10.3390/jof9020168, 36836283 PMC9960696

[ref46] Vaccaro A. Cooper J. F. Vazquez-Rodriguez A. Badali H. Kean R. Ramage G. . (2026). Candidozyma auris and the Perfect storm of fungal pathogenicity: adaptation, persistence, and resistance. J. Fungi (Basel) 12. doi: 10.3390/jof12040247, 42042342 PMC13117476

[ref47] Wei Y. Wang J. Tang N. Lin Z. Li W. Xu Y. . (2025). Caspofungin paradoxical growth in *Candida albicans* requires stress pathway activation and promotes unstable echinocandin resistance mediated by aneuploidy. Front. Cell. Infect. Microbiol. 15:1618815. doi: 10.3389/fcimb.2025.1618815, 40989191 PMC12450983

[ref48] Wek R. C. (2018). Role of eIF2alpha kinases in translational control and adaptation to cellular stress. Cold Spring Harb. Perspect. Biol. 10. doi: 10.1101/cshperspect.a032870 PMC6028073 29440070

[ref49] WHO (2022). WHO Fungal Priority Pathogens List to Guide Research, Development and Public Health Action. Geneva: World Health Organization.

[ref50] Yang F. Gritsenko V. Slor Futterman Y. Gao L. Zhen C. Lu H. . (2021a). Tunicamycin potentiates antifungal drug tolerance via aneuploidy in *Candida albicans*. MBio 12:e0227221. doi: 10.1128/mBio.02272-21, 34465026 PMC8406271

[ref51] Yang F. Kravets A. Bethlendy G. Welle S. Rustchenko E. (2013). Chromosome 5 monosomy of *Candida albicans* controls susceptibility to various toxic agents, including major antifungals. Antimicrob. Agents Chemother. 57, 5026–5036. doi: 10.1128/AAC.00516-13, 23896475 PMC3811469

[ref52] Yang F. Lu H. Wu H. Fang T. Berman J. Jiang Y. Y. (2021b). Aneuploidy underlies tolerance and cross-tolerance to drugs in *Candida parapsilosis*. Microbiol. Spectrum 9:e0050821. doi: 10.1128/Spectrum.00508-21, 34612700 PMC8510177

[ref53] Yang F. Scopel E. F. C. Li H. Sun L. L. Kawar N. Cao Y. B. . (2023). Antifungal tolerance and resistance emerge at distinct drug concentrations and rely upon different aneuploid chromosomes. MBio 14:e0022723. doi: 10.1128/mbio.00227-23, 36877011 PMC10127634

[ref54] Zhang Z. H. Sun L. L. Fu B. Q. Deng J. Jia C. L. Miao M. X. . (2024). Aneuploidy underlies brefeldin A-induced antifungal drug resistance in *Cryptococcus neoformans*. Front. Cell. Infect. Microbiol. 14:1397724. doi: 10.3389/fcimb.2024.1397724, 38966251 PMC11222406

[ref55] Zheng L. Xu Y. Guo L. (2024a). Unveiling genome plasticity as a mechanism of non-antifungal-induced antifungal resistance in *Cryptococcus neoformans*. Front. Microbiol. 15:1470454. doi: 10.3389/fmicb.2024.1470454, 39564485 PMC11573520

[ref56] Zheng L. Xu Y. Wang C. Guo L. (2024b). Ketoconazole induces reversible antifungal drug tolerance mediated by trisomy of chromosome R in *Candida albicans*. Front. Microbiol. 15:1450557. doi: 10.3389/fmicb.2024.1450557, 39139375 PMC11319258

